# Fluconazole (Diflucan®)

**DOI:** 10.1155/S1064744995000676

**Published:** 1995

**Authors:** Lisa M. Hollier, Susan M. Cox

**Affiliations:** Department of Obstetrics and Gynecology University of Texas Southzestern Medical Center 5323 Harry Hines Boulevard Dallas TX 75235 USA


Infectious Diseases in Obstetrics and Gynecology 3:222–225 (1995)
© 1996 Wiley-Liss, Inc.

### Fluconazole (Diflucan®)

**Lisa M. Hollier and Susan M. Cox**
*Department of Obstetrics and Gynecology, University of Texas Southwestern Medical Center, Dallas, TX*

Fluconazole (Diflucan®, Roerig, New York, NY) was recently developed for the treatment of systemic and surface fungal infections. It is an azole in the same family as ketoconazole and itraconazole. Fluconazole has several advantages over the other antifungal drugs including the option of oral therapy. The side-effect profile is minimal. Studies have shown it to be efficacious for the treament of vaginal yeast infections in a single oral dose.

### STRUCTURE AND DERIVATION

Fluconazole [(difluoro-2,4-phenyl)-2-bis((1H-triazole-1,2)-4-yl-1)-1,3-propanol-2] is an azole antifungal agent. The imidazole, i.e., ketoconazole, has been modified by the replacement of the imidazole ring with a triazole ring, addition of a second triazole ring, and substitution of a difluorophenyl moiety for a dichlorophenyl moiety. These changes provide more selective inhibition of fungal enzyme systems, greater metabolic stability, and enhanced water solubility.[1]

### MECHANISM OF ACTION

Fluconazole interferes with the cytochrome P-450-dependent enzyme C-14a-demethylase, which is responsible for production of ergosterol, the major component of the fungal cell membrane. The disruption of ergosterol synthesis causes structural and functional changes in the membrane which predispose the fungus to osmotic and immune-mediated damage and interfere with cell adherence.[2] The triazole derivatives bind to the cytochrome P-450 enzyme with greater specificity than their predecessors. Additional antifungal activity may be accounted for by the azole inhibition of cytochrome *c* oxidative and peroxidative enzymes, leading to increased intracellular peroxidase.[3–5]

### PHARMACOKINETICS

Fluconazole is water-soluble and thus is readily absorbed from the gastrointestinal tract. The peak plasma concentration in humans, which is reached 2 h after dosing, is 2.44–3.58 μg/ml after a 150-mg dose.[6] The presence of food does not affect the absorption of fluconazole but delays the time to maximum serum concentration until 4 h after dosing.[7] The bioavailability of fluconazole is not affected by gastric pH.[7,8] The amount of fluconazole absorbed is linearly proportional to the dose, and urinary excretion data indicate that the oral bioavailability of fluconazole is 80–90%.

Fluconazole has a volume of distribution that closely approximates total body water, 0.6–0.8 l/kg.[6] Roughly 10% of fluconazole is protein-bound in humans.[9] This low degree of binding facilitates the transfer of the drug into the central nervous system regardless of the presence of inflammation of the meninges.[10–12] Penetration of the drug into the skin and nails has been demonstrated in humans.[13] Houang et al.[6] found fluconazole to be persistent in vaginal secretions with concentrations above the MIC for *Candida albicans* for at least 72 h after a single 150-mg oral dose.

Very little metabolism of fluconazole occurs; the vast majority of it is eliminated intact through the kidney. Humphrey et al.[9] detected 3 metabolites of fluconazole in the urine of treated dogs and mice, but they comprised only 4% of the total administered drug. Two primary mechanisms influence the elimination. First, the drug is rapidly filtered as a result of the small amount of protein binding. Fluconazole is then extensively reabsorbed by the tubules, explaining its long half-life.[14]

Caution must be exercised in the administration of fluconazole to patients with impaired renal func-

Address correspondence/reprint requests to Dr. Lisa M. Hollier, Department of Obstetrics and Gynecology, University of Texas Southwestern Medical Center, 5323 Harry Hines Boulevard, Dallas, TX 75235.

Received November 10, 1995
Accepted November 27, 1995

tion. Several authors have reported a positive correlation of the drug half-life with decreasing renal function.[15] In patients with normal renal function, the half-life is 31 h. This half-life is increased to 98 h in patients with creatinine clearances of $<20$ ml/min. The manufacturer, therefore, recommends a decreased dose in patients with renal insufficiency.

### SIDE EFFECTS AND INTERACTIONS

The side-effect profile of fluconazole is favorable. Only 8.9% of the patients treated with a single 150-mg oral dose experienced adverse effects considered to be "possibly related" or "definitely related" to the drug.[16] The most common complaint was nausea (3.3%), followed by headache (1.2%), abdominal pain (0.8%), dizziness (0.7%), and heartburn or indigestion (0.7%). The adverse effects outweighed the benefit of treatment in 0.1% of patients receiving the single dose.[16]

Asymptomatic elevations of the liver transaminases have been reported in approximately 3% of individuals receiving treatment.[17] Rare fatal cases of Stevens-Johnson syndrome[18] and hepatic failure[19] have been reported in patients using fluconazole.

Because of the negligible level of hepatic metabolism, fluconazole would be expected to have fewer drug interactions than its azole predecessors. In a study of 18 healthy male volunteers, the concomitant administration of fluconazole and rifampin resulted in a 22% decrease in fluconazole's half-life.[20] These data suggest that the dose of fluconazole should be increased in those patients receiving both drugs.

The cyclosporin concentrations were significantly increased in renal transplant patients who were also treated with daily fluconazole. Torregrosa et al.[21] observed 3 patients who required at least a 50% reduction in cyclosporin dosing to maintain therapeutic concentrations. Lazar and Wilner,[22] however, found no statistically significant changes in cyclosporin plasma concentrations in bone-marrow transplant patients who were given single or multiple doses of fluconazole in addition to their stable doses of cyclosporin. These differences may occur due to the renal elimination of fluconazole. It would seem prudent to monitor cyclosporin levels in patients receiving both drugs.

The coadministration of fluconazole and tolbutamide resulted in a significant increase in the mean serum concentration of tolbutamide.[22] The administration of fluconazole has also been noted to cause an increase in serum concentrations of theophylline.[23]

A prolongation of the prothrombin time was observed in healthy volunteers receiving both warfarin and fluconazole.[22] This increase in warfarin activity may necessitate changes in the anticoagulant dose. Similarly, patients receiving both phenytoin and fluconazole were noted to have a significantly increased area under the curve (AUC) for phenytoin.[22,24]

Lazar and Wilner[22] found the pharmacokinetics of oral contraceptives to be unaffected clinically by the administration of fluconazole. Fluconazole is considered a pregnancy category C drug by the FDA; however, Inman and colleagues[25] found no demonstrably harmful effect on pregnancy or its outcome.

### SPECTRUM OF ANTIFUNGAL ACTIVITY

Fluconazole is effective in the treatment of both superficial and invasive fungal infections. There is a poor correlation between in vitro and in vivo azole activity against many fungal pathogens. Therefore, for the evaluation of the effectiveness of fluconazole, animal models have been used. Susceptible conditions include aspergillosis,[26] coccidioidomycosis,[27] histoplasmosis,[28–30] cryptococcosis,[26] blastomycosis,[29] systemic phaeohyphomycosis,[31] and candidiasis.[32–34]

### CLINICAL APPLICATIONS

Multiple clinical trials have compared the efficacy of single-dose fluconazole with topical antifungal regimens for vaginal infections. Van Heusden and coworkers[32] conducted a multicenter trial and found no differences in the clinical and mycological efficacy between fluconazole and 500-mg intravaginal clotrimazole. Moreover, a majority of patients preferred oral to intravaginal treatment. Osser et al.[33] compared econazole vaginal tablets with single 150-mg oral-dose fluconazole. At the 28–35-day follow-up, the women treated with fluconazole had a significantly higher clinical or microbiological cure rate than those given econazole.

Sobel and colleagues[34] conducted a multicenter study comparing the efficacy of single-dose 150-mg oral fluconazole with 7-day 100-mg clotrimazole vaginal tablets. At the 14-day evaluation, clinical

**TABLE 1. Costs of selected antifungal agents**

| Drug | Regimen | Cost for total treatment |
|---|---|---|
| Mycelex®[a] (clotrimazole) | Vaginal suppositories × 7 days | $9.99 |
| Gynelotrimin®[b] (clotrimazole) | Vaginal cream × 7 days | $13.33 |
| Diflucan®[c] (fluconazole) | Single 150-mg tablet | $13.70 |
| | Three 50-mg tablets | $26.25 |
| Monistat®[d] (miconazole) | Vaginal cream × 7 days | $24.72 |
| Clotrimazole | Vaginal tablets × 7 days | $24.85 |
| Terazol®[e] (terconazole) | Vaginal suppositories × 3 days | $32.68 |

[a]Miles Consumer, Elkhart, IN.
[b]Schering, Kenilworth, NJ.
[c]Roerig, New York, NY.
[d,e]Ortho, Raritan, NJ.

cure or improvement was noted in 94% of the fluconazole-treated and 97% of the clotrimazole-treated patients. At the 35-day evaluation, 75% of both groups remained clinically cured. Just over 50% of each group were considered therapeutic cures. In both treatment groups, the patients with histories of recurrent vaginitis were significantly less likely to respond clinically and mycologically.

### COST

Table 1 presents the retail costs to the patient. These costs will vary depending on the geographic location.

### SUMMARY

The modified structure of fluconazole gives it several advantages over its predecessors, including excellent oral bioavailability and an improved side-effect profile. In a single oral dose of 150 mg, fluconazole is efficacious against candidal vaginitis.

### REFERENCES

1. Richardson K, Cooper K, Marriott MS, Tarbit MH, Troke PF, Whittle PJ: Design and evaluation of a systemically active agent, fluconazole. Ann NY Acad Sci 544:4–11, 1988.
2. Ghannoum MA, Filler SG, Ibrahim AS, Fu Y, Edwards JE Jr: Modulation of interactions of *Candida albicans* and endothelial cells by fluconazole and amphotericin B. Antimicrob Agents Chemother 36:2239–2244, 1992.
3. Denollin S, Vanbelle H, Goossens F, et al.: Cytochemical and biochemical studies of yeasts after in vitro exposure to miconazole. Antimicrob Agents Chemother 11:500–513, 1977.
4. Shigematsu ML, Uno J, Arai T: Effect of ketoconazole on isolated mitochondria from *Candida albicans*. Antimicrob Agents Chemother 21:919–924, 1982.
5. Uno J, Shigematsu ML, Aria T: Primary site of action of ketoconazole on *Candida albicans*. Antimicrob Agents Chemother 21:912–918, 1982.
6. Houang ET, Chappatte O, Byrne D, Macrae P, Thorpe J: Fluconazole levels in plasma and vaginal secretions of patients after a 150-mg single oral dose and rate of eradication of infection in vaginal candidiasis. Antimicrob Agents Chemother 34:909–910, 1990.
7. Washton H: Review of fluconazole: A new triazole antifungal agent. Diagn Microbiol Infect Dis 12:229S–233S, 1989.
8. Blum RA, Wilton JH, Hilligoss DM, Gardner MJ, Henry EB, et al.: Effect of fluconazole on the disposition of phenytoin. Clin Pharmacol Ther 49:420–425, 1991.
9. Humphrey MJ, Jevons S, Tarbit MH: Pharmacokinetic evaluation of UK-49,858, a metabolically stable triazole antifungal drug, in animals and humans. Antimicrob Agents Chemother 28:648–653, 1985.
10. Foulds G, Wajszczuk C, Weidler DJ, Barg DC, Gibson P: Steady state parenteral kinetics of fluconazole in man. Ann NY Acad Sci 544:427–430, 1988.
11. Foulds G, Brennan DR, Wajszczuk C, Cantanzaro A, Garg DC, et al.: Fluconazole penetration into cerebrospinal fluid in humans. J Clin Pharmacol 28:363–366, 1988.
12. Tucker RM, Williams PL, Arathoon EG, Levine BE: Pharmacokinetics of fluconazole in cerebrospinal fluid and serum in human coccidioidal meningitis. Antimicrob Agents Chemother 32:369–373, 1988.
13. Haneke E: Fluconazole levels in human epidermis and blister fluid. Br J Dermatol 123:273–277, 1990.
14. Debruyne D, Ryckelynck JP: Clinical pharmacokinetics of fluconazole. Clin Pharmacokinet 24:10–27, 1993.
15. Toon S, Ross CE, Gokal R, Rowland M: An assessment of the effects of impaired renal function and haemodialysis on the pharmacokinetics of fluconazole. Br J Clin Pharmacol 34:75–78, 1990.
16. Phillips RJM, Watson SA, McKay FF: An open multicentre study of the efficacy and safety of a single dose of fluconazole 150 mg in the treatment of vaginal candidiasis in general practice. Br J Clin Pract 44:219–222, 1990.
17. DeWit S, Goossens H, Weerts D, Clumeck N: Comparison of fluconazole and ketoconazole for oropharyngeal candidiasis in AIDS. Lancet 1:746–748, 1989.
18. Powderly WG, Saag MS, Cloud GA, et al.: A controlled trial of fluconazole or amphotericin B to prevent relapse of cryptococcal meningitis in patients with the acquired immunodeficiency syndrome. N Engl J Med 326:793–798, 1992.
19. Jacobson MA, Hanks DK, Ferrell LD: Fatal acute hepatic injury arising during fluconazole therapy. Arch Intern Med 154:102–104, 1994.
20. Apseloff G, Hilligoss DM, Gardner MJ, et al.: Induction of fluconazole metabolism by rifampin: In vivo study in humans. J Clin Pharmacol 31:358–361, 1991.
21. Torregrosa V, De la Torre M, Campistol JM, et al.: Interaction of fluconazole with cyclosporin A. Nephron 60:125–126, 1992.
22. Lazar JD, Wilner KD: Drug interactions with fluconazole. Rev Infect Dis 12:s327–333, 1990.
23. Konishi H, Morita K, Yamaji A: Effect of fluconazole on theophylline disposition in humans. Eur J Clin Pharmaol 46:309–312, 1994.
24. Blum RA, Wilton JH, Hilligoss DM, et al.: Effect of fluconazole on the disposition of phenytoin (abstract). Clin Pharmacol Ther 47:182, 1990.
25. Inman W, Pearce G, Wilton L: Safety of fluconazole in the treatment of vaginal candidiasis. A prescription-event monitoring study, with special reference to the outcome of pregnancy. Eur J Clin Pharmacol 46:115–118, 1994.
26. Troke PF, Andrews RJ, Marriott MS, et al.: Efficacy of fluconazole (UK-49,858) against experimental aspergillosis and cryptococcosis in mice. J Antimicrob Chemother 19:663–670, 1987.
27. Graybill JR, Sun SH, Ahrens J: Treatment of murine coccidioidal meningitis with fluconazole (UK49,858). J Med Vet Mycol 24:113, 1986.
28. Kobayashi GS, Travis SJ, Medoff G: Comparison of fluconazole and amphotericin B in treating histoplasmosis in immunosuppressed mice. Antimicrob Agents Chemother 31:2005–2006, 1987.
29. Smith E, Franzmann M, Mathiesen LR: Disseminated histoplasmosis in a Danish patient with AIDS. Scand J Infect Dis 21:573–577, 1989.
30. Dismukes WE, Bradsher RW, Cloud GC, et al.: Itraconazole therapy for blastomycosis and histoplasmosis. NIAID Mycoses Study Group. Am J Med 93:489–497, 1992.
31. Dixon DM, Polak A: In vitro and in vivo drug studies with three agent of central nervous system phaeohyphomycosis. Chemotherapy 33:129–140, 1987.
32. Van Heusden AM, Merkus HMWM, Euser R, Verhoeff A: A randomized, comparative study of a single oral dose of fluconazole versus a single topical dose of clotrimazole in the treatment of vaginal candidosis among general practitioners and gynaecologists. Eur J Obstet Gynaecol Reprod Biol 55:123–127, 1994.
33. Osser S, Haglund A, Westrom L: Treatment of candidal vaginitis. A prospective randomized investigator-blind multicenter study comparing topically applied econazole with oral fluconazole. Acta Obstet Gynaecol Scand 70:73–78, 1991.
34. Sobel JD, Brooker D, Stein GE, et al.: Single oral dose fluconazole compared with conventional clotrimazole topical therapy of *Candida* vaginitis. Am J Obstet Gynecol 172:1263–1268, 1995.
